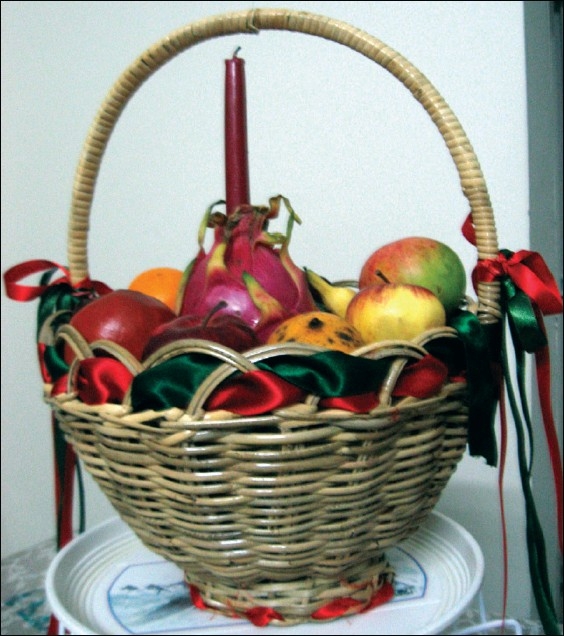# A basket full!

**Published:** 2009

**Authors:** Sanjeev V. Thomas

**Affiliations:** Professor of Neurology, SCTIMST, Trivandrum - 695 011, India. E-mail: sanjeev.v.thomas@gmail.com